# Highly Productive Laser Annealing Manufacturing Method Using Continuous Blue WBC (Wavelength Beam Combining) Technique

**DOI:** 10.3390/ma17225399

**Published:** 2024-11-05

**Authors:** Mitsuoki Hishida, Naohiko Kobata, Kentaro Miyano, Masaki Nobuoka, Tatsuya Okada, Takashi Noguchi

**Affiliations:** 1 Panasonic Connect Co., Ltd., 3-1-1 Inazu-cho, Toyonaka City 561-0854, Osaka, Japan; 2 University of the Ryukyus, Nishihara 903-0213, Okinawa, Japan; tokada@tec.u-ryukyu.ac.jp (T.O.);

**Keywords:** direct diode laser, wave beam combining, blue diode, blue laser annealing, excimer laser annealing, polysilicon, thin-film transistor

## Abstract

Blue laser annealing can be used to obtain a high-mobility thin-film transistor (TFT) through a laser annealing (i.e., LTPS: low-temperature Poly-Si) process. However, the laser annealing process’s low productivity (as well as high cost) is an issue because the high output power of blue lasers still needs to be addressed. Therefore, productivity can be improved if blue laser energy is efficiently supplied during the laser annealing process using a continuous wave laser instead of a conventional pulsed excimer laser. We developed a blue laser light source (440 ± 10 nm) using the wavelength beam combining (WBC) method, which can achieve a laser power density of 73.7 kW/cm^2^. In this semiconductor laser, when the power was increased s by 2.9 times, the laser scanning speed was increased by 5.0 times, achieving twice the productivity of conventional lasers. After laser annealing, the size of the crystal grains varied between 2 and 15 μm, resulting in a crystallization rate of 100% by Raman scattering rsult and low resistivity of 0.04 Ωcm. This increase in production capacity is not an arithmetic increase with increased power but a geometric production progression.

## 1. Introduction

In a flat-panel display using low-temperature polysilicon thin-film transistors (LTPS TFTs) as the backplane, the excimer laser annealing (ELA) process with a wavelength of 308 nm (XeCl) is used to form a polysilicon (p-Si) film by crystallizing an amorphous silicon (a-Si) film to improve carrier mobility [1]. Many reports have been made on laser annealing (LA) using the CW mode to further improve carrier mobility. Blue laser diode annealing (BLDA) technology is a new approach that uses a semiconductor blue laser instead of the conventional ELA (249/308 nm) [2,3,4,5]. BLDA is a promising technology that utilizes the blue laser, capable of penetrating deeper into the a-Si film, thereby increasing the grain size of crystallized Si by uniformly controlling the temperature going into the thin a-Si film during laser annealing. However, because it is difficult to increase the output power of blue or green laser light sources compared with conventional excimer lasers, practical mass production will be a challenge in the future [6,7,8,9].

Wavelength beam combining (WBC) technology improves the laser’s beam parameter product (BPP) in industrial lasers for welding and cutting metals. It increases the laser power density to improve the processing accuracy and energy efficiency [6,10]. WBC technology provides high beam quality with improved BPP [=(the product of the radius of the beam waist) × (the full width at half-maximum of the divergence angle of the beam)]. The widely utilized SBC technology is favored for its simplicity of construction, using lenses to concentrate light. However, at higher blue laser outputs, precisely above 1.5 kW, the beam parameter product (BPP) is reported to range from 17 to 39 mm mrad [6,11,12]. This contrasts markedly with the BPP of 3.2 mm mrad at 2.16 kW for a high-power blue laser employing a WBC structure [13]. As the laser light source must be transmitted from the source to the processing site through a fiber or similar medium, the BPP should readily enable light to enter the fiber’s core diameter and possess a significant focal depth, which is essential for a high-density laser. A lower BPP value facilitates entry into the fiber and results in a greater laser power density. Consequently, the laser power density of the WBC can be augmented by 10 to 20 times relative to that of spatial beam-combined lasers [6].

The laser annealing process increases the heat transfer rate of the a-Si film as the laser power density increases [14]. If the heat transfer rate is faster, the loss of laser energy can be reduced, and the laser irradiation time can be shortened, thereby shortening the process time and increasing productivity [15].

Panasonic’s blue laser light source, with an output of 2.16 kW and a BPP of 3.2 mm mrad, utilizes WBC technology to reduce the BPP, enabling the generation of high-density laser light [13]. Consequently, this laser light source was compared with the conventional ELA laser light source, which has an output of 3.6 kW and a processing capacity of 26.4 sheets/hour for a G6 size substrate (=7.3 sheets/hour/kw) [16,17]. This study conducted experiments for actual films to compare the production capacity of the laser annealing process using the blue laser light source (440 ± 10 nm) formed by the CW-WBC technology.

## 2. Experimental

### 2.1. WBC Technologies

**Figure 1:**
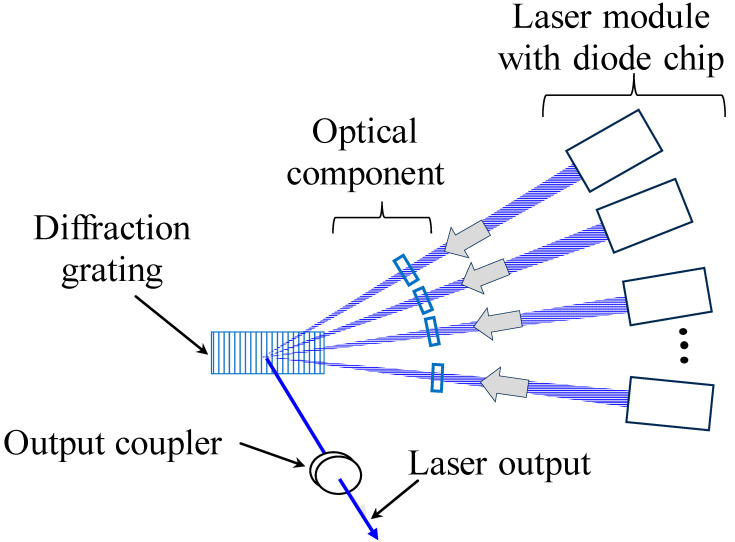
Schematic illustration of wavelength beam combining [6,10,18].

Figure 1 illustrates the WBC technology used for the high-density laser light source. The setup primarily comprises multiple laser modules, including a light source, optical components, diffraction gratings, and output couplers. The laser light source used in the WBC technologies uses slightly different wavelengths. The WBC light source utilized in this study emits wavelengths within the 430–450 nm range. Multiple laser beams emitted from bar-type LD modules overlap through the diffraction grating. Each beam is refracted at different angles at the grating owing to the slight differences in their wavelengths. These beams can then be focused onto a single beam, resulting in higher power output while maintaining beam quality comparable to that of a single beam. This technology, which concentrates multiple lights into a single beam, utilizes the phenomenon of light dispersion through a prism. WBC technology utilizes these phenomena, with light entering from the reverse direction [18]. We confirmed that this system achieves maximum beam brightness, and that the BPP (beam parameter product) output is 2.0 milli-radian [6,10].

### 2.2. Experimental Methods and Apparatus

A laser annealing experiment examined the relationship between power density and productivity by irradiating a blue laser beam onto an actual sputtered a-Si film substrate.

**Figure 2:**
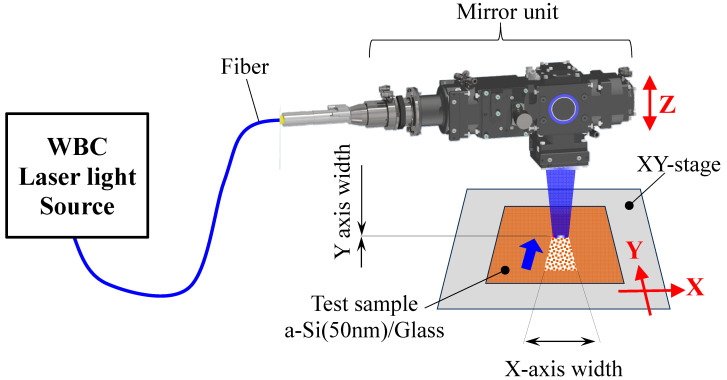
Appearance model diagram of equipment with line laser processing [15].

For the substrate sample used for laser annealing, an a-Si film with a thickness of 50 nm was deposited on Eagle XG glass (Corning Incorporated, NY.USA), which is commonly used in the LTPS process, based on a heavy Phos.-doped Si target (standard: 0.01–0.02 Ωcm) using the RF sputtering method. The laser beam, enhanced by a laser light source component using WBC technology in the oscillator, was transmitted to an optical head unit through a fiber. The length of the line lasers (X-axis direction) was approximately 4.0 mm, and the width in the laser scanning direction (Y-axis) was adjusted from the minimum width of 43–151 μm to meet the power density change conditions. The a-Si film on the glass sample was fixed on a stage, and laser annealing was performed as the sample stage moved at an arbitrary speed along the Y-axis. Figure 2 shows a schematic overview of the line laser annealing equipment [15].

**Figure 3:**
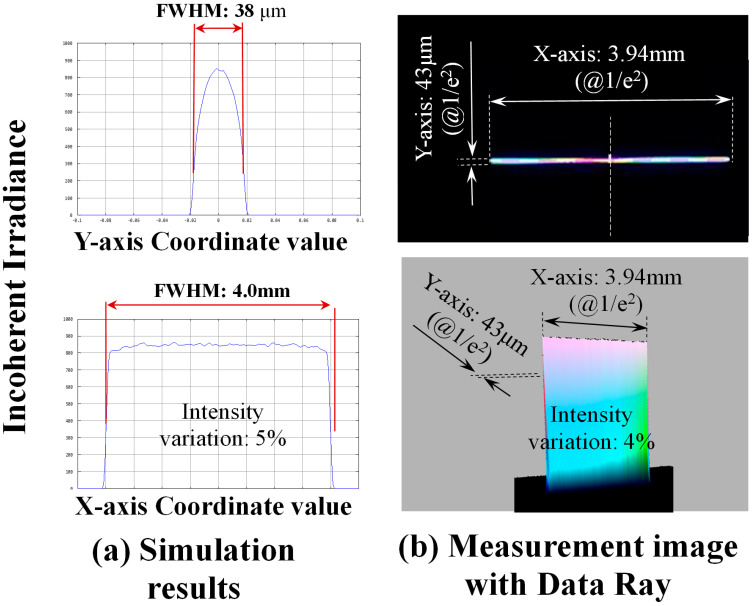
Beam profile of Condition E [15].

A CW laser light source with an output of 124 W was kept constant under all conditions. The width in the laser scanning direction (Y-axis) was changed to 151, 111, 67, 47, and 43 μm at values of 1/e^2^ by changing the stage height. The conditions were defined as A, B, C, D, and E, respectively, which were used as conditions for changing the power density. The Y-axis width value was determined using a Data Ray measurement device (Win Cam D-LCM—1 CMOS Beam Profiler, Ver.8.0D92, DataRay Inc., Redding, CA, USA). Although this measurement device has a minimum resolution of 5.5 μm, the measured values of 1/e^2^ were applied as they were. Figure 3a shows the analysis results when the analysis value of the width (Y-axis) was 38 μm and the X-axis was 4.0 mm at FWHM in the laser scanning direction [15]. The measurement results of 1/e^2^ by Data Ray showed that the Y-axis was indicated at 43 μm and the X-axis at 3.94 mm, as shown in Figure 3b [15].

In the simulation results in Figure 3a, 5% fluctuation was observed in the X-axis direction. Still, the power meter measurement results shown in Figure 3b showed that the laser fluctuation in the X-axis direction was within 4%. However, monitoring the laser output fluctuation with a power meter before the actual process is insufficient. Therefore, we performed laser annealing experiments to ensure power consistency under various laser power conditions. Each measured line laser spot length ranging from 3.94 to 3.50 mm was used to evaluate the characteristics of the high-density output. Therefore, to apply this line laser size to a large substrate size, such as G6 with a length of 1500 mm, adjusting the X-axis length and scanning speed is necessary to accommodate different substrate sizes, ensuring in-plane uniformity and production efficiency.

The laser beam profile had a Gaussian shape in the laser scanning direction and did not have a top hat shape. However, as the Gaussian shape of the profile was scanned perpendicular to the laser scanning direction, the energy provided in the laser annealing process was assumed to be constant in the scanning direction. The power density was calculated as follows: power density = [(laser output power)/ (laser irradiation area at 1/e^2^)]. In Condition A, the laser light source output was 124 W, the Y-axis width was 151 μm, the X-axis width was 3.50 mm, and the power density was calculated to be 23.4 kW/cm^2^. The power density values under conditions B–E were calculated using the same method, and the experiments were conducted with power density values of 31.4, 50.6, 67.0, and 73.7 kW/cm^2^ for conditions B, C, D, and E, respectively. Table 1 lists the Y-axis widths and associated laser power density values for conditions A–E.

## 3. Results

### 3.1. Experimental Results

Experiments were conducted under conditions A, B, C, D, and E at different laser power densities and scanning speeds. All the experiments were conducted using a laser power of 124 W and an a-Si substrate for irradiation (a-Si film deposited by RF sputtering with a thickness of 50 nm (±4 nm) was formed on an Eagle XG glass substrate). The laser scanning speed varied from 80 to 1000 mm/s depending on the laser density, and processing was performed under 39 conditions.

A sample with a crystallization rate of 95% or more by Raman spectrum evaluation was indicated as p-Si, a sample with a crystallization ratio of 20–95% was indicated as microcrystalline silicon (μc-Si), and a sample with a crystallization ratio of 20% or less was indicated as roughly a-Si. Microcrystalline conditions are the exact boundary between the conditions for the transition from amorphous to crystalline. A Raman spectrometer was used to evaluate the crystallization after laser annealing. A Raman spectrometer with an excitation light source of the laser wavelength 488.0 nm/measurement area diameter of 1 μmφ was used to measure crystallinity. Waveform separation was performed for the two peaks, the crystalline phase near 510–520 cm^−1^ and the amorphous phase near 480 cm^−1^, to analyze the crystallization rate using the Lorentzian function. The Xc crystallization ratio is X_c_ = S_c_/(S_c_ + S_a_) × 100%, where S_c_ and S_a_ are the peak areas for single-crystalline and amorphous phases, respectively [19,20].

**Figure 4:**
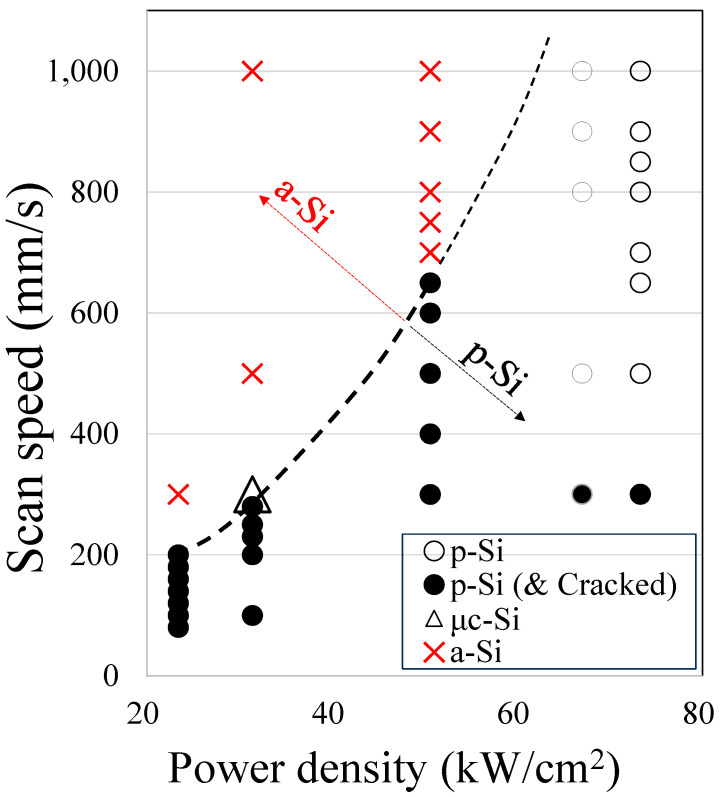
Evaluation of crystallization/substrate cracking at each power density and scanning speed.

**Figure 5:**
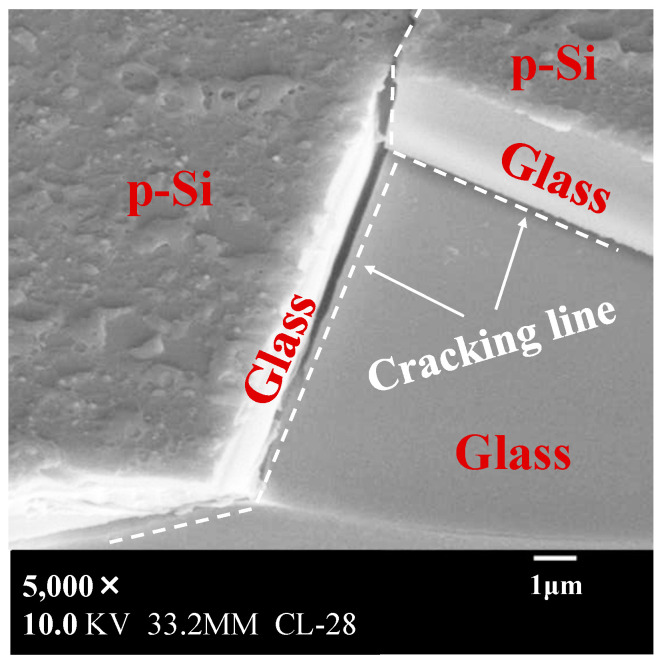
Top view of the cracked area after laser annealing. (In addition to the annealed p-Si on the surface, approximately 5 μm peeled off from the glass surface, revealing a portion of the glass interior in the lower right of the picture).

In Figure 4, the condition of the a-Si film under laser annealing is shown as a red cross, where the films were crystallized to μc-Si in triangles and where the films were successfully crystallized higher than or close to 95% in circles and black dots. In this experiment, cracks occurred in the glass substrate after laser annealing; the results are indicated by white and black circles. The black dots indicate the crystallization results achieved with cracked substrates. Figure 5 shows a top view of the cracked area in the glass after laser annealing. In addition to vertical glass cracking, p-Si was peeled off along with the glass substrate at a depth of approximately 5 μm [21].

Under Condition A, when the laser scanning speed was 300 mm/s, crystallization did not occur, and the silicon film remained in the amorphous phase. However, crystallization occurred when the laser scanning speed was reduced to 200 mm/s. The fastest scanning speed for crystallization under these conditions was 200 mm/s. Unfortunately, all the glass substrates cracked while the films crystallized. The laser beam’s energy was transmitted to the substrate after crystallizing the a-Si, and cracks occurred because the applied energy was greater than required for the glass’s thermal expansion. The CW blue laser did not contribute efficiently to the laser annealing process.

In Condition B, when the laser scan speed was 500 mm/s, the a-Si did not crystallize and remained in the amorphous phase. However, when the scanning speed was changed to 300 mm/s, the exposed Si films were found to be µc-Si. Crystallization was performed when the scanning speed was slower than 280 mm/s. Under Condition B, the fastest laser scan speed for crystallization was 280 mm/s, which was quicker than that under Condition A.

On the other hand, cracks were observed when the speed was 280 mm/s or slower. However, all glass substrates cracked while the films crystallized. Even in Condition B, the CW blue laser energy could not efficiently contribute to the laser annealing process.

In Condition C, at a faster laser scanning speed of 700 mm/s, the Si film remained in the amorphous phase without becoming p-Si. However, p-Si was obtained when the laser scan speed was 650 mm/s or slower. The fastest speed for crystallization under Condition C was when the laser scanning speed was 650 mm/s. On the other hand, substrate cracking occurred when the laser scanning speed was 650 mm/s or less. This variation in speed increased significantly from conditions B to C compared to that in conditions A to B. However, all the glass substrates cracked while the films crystallized. The CW blue laser energy could not efficiently contribute to the laser annealing process without breaking the glass substrate, even in Condition C.

In conditions D and E, p-Si was obtained for all the substrates, even when the transportation speed was increased to 1000 mm/s. This shows that the annealing process speed increased further than in Condition C. As the highest speed of the experimental equipment was limited to 1000 mm/s, the laser scanning speed for crystallization could be operated at 1000 mm/s or higher. However, in conditions D and E, when the laser scanning speed was 500 mm/s or higher, no cracks occurred on the glass substrates while achieving crystallization. However, crystallization was achieved when the laser scanning speed was 300 mm/s or lower, although the substrate was cracked.

### 3.2. Experimental Considerations

The fastest laser processing scanning speed resulting in crystallization was 200 mm/s in Condition A, 280 mm/s in Condition B, and 650 mm/s in Condition C. A comparison of these results is presented in Table 2. The power density values of conditions A, B, and C are 23.4, 31.4, and 50.6 kW/cm^2^, respectively. The power density values of Conditions B and C are 1.3 and 2.2 times, respectively, higher than that of Condition A (23.4 kW/cm^2^). On the other hand, the maximum laser scanning speed at which crystallization was achieved in the laser annealing process increased by 1.4 and 3.3 times for Conditions B and C, respectively, compared to that for Condition A. Moreover, the increase in this ratio surpassed that of the power density ratio. This result shows that the improvement in the process capability was more significant than the increase in the power density of laser annealing. Also, it was a geometric increase in productivity rather than an arithmetic increase compared to the increase in power density. Furthermore, although a-Si was crystallized in conditions A, B, and C, multiple cracks occurred on the glass substrates, as shown in Figure 5. This indicates that the CW laser did not efficiently contribute to the crystallization process. Similarly, a comparison of the energy densities shows the difference in the annealing efficiency for crystallization between conditions A, B, and C. Condition A requires an energy density of 17.7 J/cm^2^, whereas in conditions B and C, as the laser power density increases, the energy density can be reduced to 12.4 and 5.2 J/cm^2^, respectively [22].

In Condition D (67.0 kW/cm^2^), where the fastest scanning speed could not be identified, the power density increased by 2.9 times compared to the power density of Condition A (23.4 kW/cm^2^). The fastest measurable laser scanning speed under Condition D was 1000 mm/s, and the laser annealing scanning speed improved five times at this value. It can be confirmed that the laser annealing scanning capacity increased more than the power density. The enhancement in scanning speed from Condition C to D was more significant than the improvements observed from A to B or B to C. This experiment confirmed that increasing the laser power density improves the heat transfer rate, which in turn increases the scanning speed [4,14,15]. These faster scanning speeds improved the laser annealing productivity. The experimental results suggest that 4.3 and 21.4 sheets of the G6 (1500 × 1850 mm) substrates can be processed per hour using a Panasonic 400 W oscillator and Panasonic 2000 W oscillator, respectively, when the laser width is 4 mm and the scanning speed is 1000 mm/s [13]. The loading and unloading times of the substrate samples are not included in the calculations. This value indicates that the processing capacity is the same as that of conventional laser annealing equipment, which has approximately twice the laser output power at 3600 W [16,17]. This is because CW lasers do not have a laser off time similar to that of conventional pulsed laser equipment. A scanning speed of 1000 mm/s was the limit of the equipment, not the processing capacity; therefore, it can achieve even greater productivity.

Furthermore, the increase in the laser energy density, as observed in conditions D and E, helps the laser energy contribute to the laser annealing process and reduces the heat stress applied to the glass substrates, thereby reducing glass cracking [23]. A high-density laser power can reduce the risk of cracking during the annealing process of CW lasers, which do not have a cooling time similar to that of pulsed lasers [4,23]. The increase in productivity can be confirmed by the fact that there was no glass cracking, thus achieving efficient laser annealing. The no-glass cracking process is also effective for forming p-Si TFT on flexible plastic substrates with weak heat resistance and is expected to expand the market in the future [24].

Table 2 compares the four conditions and production capacities (G6 board production capacities per hour using 400 W and 2000 W oscillators). In this experiment, we used a line laser with a width of 4 mm to confirm the power density and productivity. However, with an optical design, it was possible to support large substrate sizes, such as G6. Increasing the power density in the laser annealing process under the same laser power conditions enables the fastest scanning speed of laser annealing. The energy was more efficiently used as crystallization energy as the laser energy density increased [14]. Therefore, with a laser output of 2000 W, the production capacity for the G6 size substrates can be confirmed to increase geometrically from 4.3 to 21.4 pieces per hour using a high-density laser condition.

### 3.3. Grain Size Observation by Electron Back-Scattered Diffraction (EBSD)

**Figure 6:**
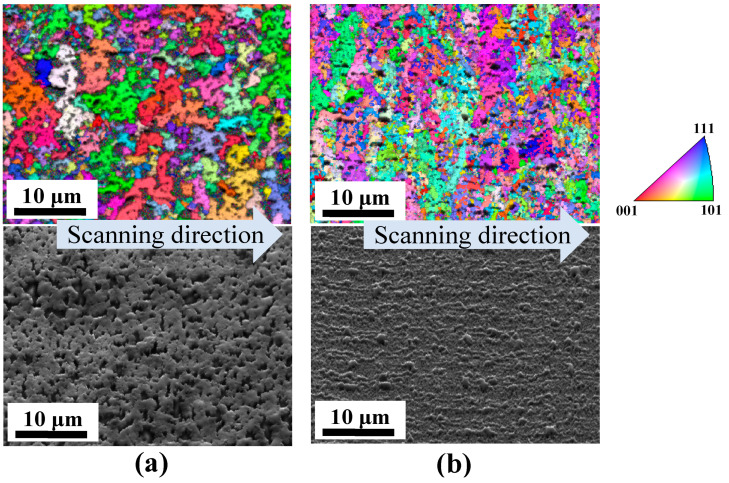
Comparison of EBSD results at two different scan speeds: (**a**) 500 mm/s; (**b**) 1000 mm /s.

EBSD using scanning electron microscopy (SEM) was conducted to determine the crystal grain size [25]. Two substrate types were assessed at scanning speeds of 500 and 1000 mm/s, utilizing the exact line laser dimensions in Condition E (laser width: 43 μm; laser length: 3.94 mm at 1/e^2^), with a laser power of 221 W. Under both conditions, the crystallization rate was 100% in the in-plane distribution, and the substrate exhibited no glass cracks. The results are shown in Figure 6a,b.

The EBSD analysis revealed that the speculated crystal grain size for both substrates ranged from 2 to 15 μm, which exceeds the grain size of typical crystalline polysilicon created with the ELA [15,26]. At a scan speed of 500 mm/s (a), partial indentations were noted, likely resulting from the melting and subsequent loss of some crystalline silicon, attributable to the high energy input at this slower speed.

Under 500 mm/s (a), the glass substrate did not crack. However, the excess energy caused silicon to disappear and dent the surface. Conversely, setting the scan speed to 1000 mm/s (b) resulted in no dents on the surface, indicating that the silicon crystallized effectively instead of vanishing. However, the EBSD results revealed a speckled black pattern in Condition b, distinct from the dent caused by the melting of silicon observed in Condition a. The hypothesis is that the lens’s shape of the line laser is related to the laser’s scanning direction, although this is under discussion.

### 3.4. Consideration of Raman Spectrum

**Figure 7:**
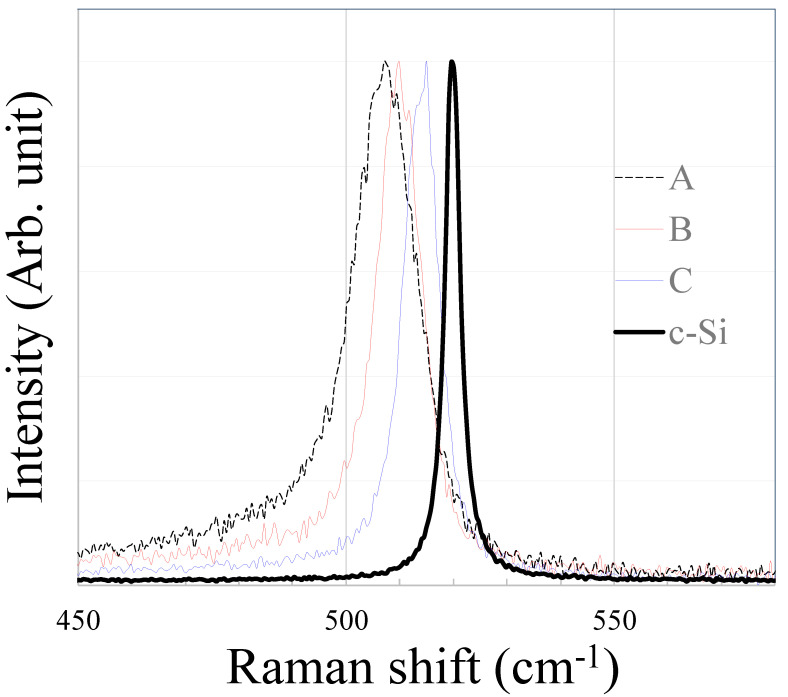
Comparison of Raman spectrum shifts at the fastest processing speed with different power densities (conditions A, B, C).

Figure 7 shows the Raman spectra of films crystallized at the highest scanning speeds of 200–650 mm/s for conditions A, B, and C. The peak values of the Raman spectrum shifts, crystallization rates, and FWHM results are listed in Table 2. Figure 7 also compares the measurement results for single-crystalline Si (c-Si). For these three substrates, the peak value of the Raman spectrum shifted to a shorter wavenumber as the power density decreased, indicating that the tensile stress of the crystallized p-Si film increased [21,27,28]. Using a high-density laser is more effective in obtaining a p-Si film with low stress under this condition, increasing the laser power density, which results in forming a p-Si film with less stress on the substrate. The FWHM value decreases as the power density increases. These results indicate that the conditions are close to producing a high-quality film similar to c-Si. Condition D could not be performed under the fastest conditions of crystallinity owing to the limited equipment capacity. Therefore, comparing these conditions with conditions A, B, and C was difficult.

### 3.5. Electrical Resistivity

Table 2 also displays the electrical resistivity, indicative of the electrical properties ascertained through the four-probe method [29]. This technique employs a probe with needle spacing set at 1 mm and a measurement interval of 3 mm. The electrical resistivity was recorded in two orientations: parallel, where the laser annealing’s scanning direction aligns with the four probes’ direction, and perpendicular, where it is rotated 90 degrees.

The minimum resistivity recorded in the parallel direction under conditions A, B, and C was 0.04 Ωcm). This value approaches the standard electrical resistivity range for sputtering targets (0.01–0.02 Ωcm). This suggests that high-quality crystallization was attained [30].

On the other hand, the electrical resistivity in the vertical direction was consistently high. This may be due to the occurrence of microcracks in the substrate, as shown in Figure 5, or the lack of uniformity of the optical lens, which resulted in non-crystallized areas along the laser scan path, as shown by the black line in the scan direction in Figure 6. Regardless, the cause is being investigated.

Our research employed a-Si film deposited through sputtering, which offers economic advantages in producing lower-temperature TFT devices. However, to densify the film post-deposition, we used dehydrogenated PECVD (plasma-enhanced chemical vapor deposition) Si film, and uniform crystallization was stably achieved without any cracks or dark lines in the scanning direction [31]. These outcomes indicate that the annealing conditions in our study necessitate additional optimization.

### 3.6. The Roughness of Laser-Crystallized Poly-Si

In the fabrication of TFTs, surface roughness following laser annealing is a critical parameter as it impacts the uniformity of the subsequently deposited film [32]. The measured roughness of the polysilicon surface under conditions C and D yielded values of Ra: 2 nm and 1 nm, respectively, within an evaluation area of 0.5 mm square, satisfying the criteria for TFT production [15].

## 4. Conclusions

Using WBC technology, a line laser was generated from a blue laser, effectively reducing the BPP for high-density applications. A laser annealing experiment was performed, and the study increased the output to a maximum of 73 kW/cm^2^ by analyzing the relationship between the line laser size and the transport speed. A comparative experiment on power density revealed enhanced productivity. Furthermore, the properties of the film post-laser-processing indicated an increase in crystal grain size while maintaining electrical resistivity, and a surface roughness of Ra: 1 nm was achieved.

Utilizing a 2.16 kW oscillator from Panasonic with a laser power density of 73.7 kW/cm^2^, one can anticipate a throughput of 21.4 sheets per hour for G6 (1500 × 1850 mm) substrates (=9.9 sheets/hour/kw) [13]. This capacity effectively increases the laser annealing processes per unit of laser output compared to conventional ELA laser annealing equipment, which typically operates at around 3.6 kW (=7.3 sheets/hour/kw). The advantage of CW lasers over traditional pulsed lasers is that they operate continuously without any downtime [25]. Laser energy utilization becomes more efficient by enhancing the energy density and enlarging the annealing area, which could reduce manufacturing costs. Additionally, Raman analysis has demonstrated that high-power lasers can produce high-quality crystalline films with minimal film stress, promising improvements for future laser annealing techniques.

## Figures and Tables

**Table 1:** Experiment parameters under the conditions A, B, C, D, and E.

	A	B	C	D	E
Power density (kW/cm^2^)	23.4	31.4	50.6	67.0	73.3
Laser power (W)	124.0				
Scanning speed (mm/s)	80–300	100–1000	300–1000		
Laser spot width [Y-axis] (mm) @1/e^2^	0.151	0.111	0.067	0.047	0.043
Laser spot length [X-axis] (mm) @1/e^2^	3.50	3.56	3.66	3.92	3.94
Spot size (cm^2^) @1/e^2^	0.0053	0.0039	0.0025	0.0019	0.0017

**Table 2:** Comparisons of four conditions and production capacities, and Raman measurement results between A, B, C, and D.

Laser Width Condition	Power Density		Maximum Scan Speed for Crystal		Energy Density		Production Capacity Per Hour in G6 Size Substrates		Peak Position (cm^−1^)	Crystallization State	Electrical Resistivity (Ωcm)		FWHM (cm^−1^)
	(kW/cm^2^)	Increase Rate from Condition A	(mm/s)	Increase Rate from Condition A	(J/cm^2^)	Increase Rate from Condition A	@400 (W) Oscillator	@2000 (W) Oscillator			Parallel	Perpendicular	
A	23.4	―	200	―	17.7	―	0.9	4.3	508	p-Si	0.04	9.5 × 10^4^	13.9
B	31.4	1.3	280	1.4	12.4	0.7	1.2	6	510	p-Si	1.15	2.3 × 10^4^	8.8
C	50.6	2.2	650	3.3	5.2	0.3	2.8	13.9	515	p-Si	0.07	2.5 × 10^6^	7.4
D	67	2.9	(1000)	(5)	3.2	0.2	4.3	21.4	513	p-Si	75	8.0 × 10^3^	9.7

## Data Availability

The original contributions presented in the study are included in the article, further inquiries can be directed to the corresponding author.
